# Protective Effect of Short-Term Genistein Supplementation on the Early Stage in Diabetes-Induced Renal Damage

**DOI:** 10.1155/2013/510212

**Published:** 2013-04-29

**Authors:** Min Ju Kim, Yunsook Lim

**Affiliations:** Department of Food and Nutrition, Kyung Hee University, Seoul 130-701, Republic of Korea

## Abstract

Hyperglycemia-induced oxidative stress has been concerned in the development of diabetic nephropathy (DN), which may cause kidney damage associated with inflammation and fibrosis. This study has been conducted to investigate the role of genistein supplementation in an acute DN state. Mice with FBG levels more than 250 mg/dL after alloxan injection (single i.p., 150 mg/kg) were considered as diabetic. Diabetic mice (DM) were further subdivided according to their FBG levels, medium-high FBG (DMMH < 450 mg/dL) and high FBG (DMH; 450 mg/dL) and were administrated by an AIG-93G diet supplemented with different doses of genistein (0, 0.025 or 0.1%). After 2 weeks' treatment, the levels of kidney malondialdehyde (MDA), blood urea nitrogen (BUN), and plasma creatinine and lipid profiles, as well as oxidative stress and inflammation-related markers, were measured (*P* < 0.05). Genistein supplementation improved levels of FBG in the DMMH groups, but not in the DMH group, regardless of the treatment dose. Moreover, the supplementation attenuated kidney oxidative stress indicated by MDA, BUN, and plasma creatinine. In addition, genistein treatment decreased inflammatory markers such as nuclear factor kappa B (p65), phosphorylated inhibitory kappa B alpha, C-reactive protein, monocyte chemotactic protein-1, cyclooxygenase-2, and tumor necrosis factor-alpha and improved oxidative stress markers (nuclear-related factor E2, heme oxygenase-1, glutathione peroxidase, and superoxide dismutase isoforms) in treatment groups, regardless of the genistein treatment dose. Furthermore, genistein supplementation inhibited the fibrosis-related markers (protein kinase C, protein kinase C-beta II, and transforming growth factor-beta I) in the DN state. However, 0.1% genistein supplementation in diabetes with high FBG levels selectively showed a preventive effect on kidney damage. These results suggest that genistein might be a good protective substance for DN through regulation of oxidative stress and inflammation. In particular, genistein is more efficient in diabetes patients with medium-high blood glucose levels. Finally, it is required to establish the beneficial dosage of genistein according to blood glucose levels.

## 1. Introduction

Diabetes mellitus (DM) is a major endocrine-metabolic disorder that is associated with chronic hyperglycemia by disturbance in carbohydrate, protein, and lipid metabolism. According to the WHO (World Health Organization), the world prevalence of diabetes has been increasing explosively from 171 million in 2000 to an assumed 366 million in 2030 [1]. As DM have severe health consequences, it gives rise to diabetic complications including retinopathy, neuropathy, and nephropathy. About 20%–40% of diabetic patients suffer from diabetic nephropathy (DN), which is characterized by end-stage renal disease [2]. DN has been implicated in several mechanisms by hyperglycemia, which may simulate overproduction of reactive oxygen species (ROS). ROS play a crucial role in generation of oxidative stress and several inflammatory responses [3, 4] that trigger cellular dysfunction and progression of kidney fibrosis. Indeed, the response may be upregulated by ROS-related activation of transcription factors and their downstream genes. This fact suggests that the mechanism of several transcription factors is implicated in hyperglycemia-mediated expression of genes involved in DN [5]. Recently, it has become increasingly acknowledged that NF*κ*B generally works with other transcription factors [6, 7], such as nuclear related factor E2 (Nrf2) [8]. DN condition is expected to bring out diverse synergistic effects at the transcriptional level [6]. NF*κ*B induced by oxidative stress is one of the most critical transcriptional regulatory factors that control the expression of a large number of genes involved in inflammatory response, including cytokines, chemokines, growth factors, and adhesion molecules [9]. It mediates damages in extracellular matrix, glomerulosclerosis, and renal failure, thus stimulating the development of DN. Recently, an increase in NF*κ*B activation has been observed in DM patients [10, 11] and in DN animals [12]. In contrast to the inflammatory action of NF*κ*B, Nrf2 is responsible for the defense system against oxidative stress [13, 14] and inflammation [8] by regulation of phase II detoxifying enzymes and redox-related antioxidant proteins [15]. It is known that activation of Nrf2 and upregulation of its downstream antioxidant genes in hyperglycemic condition were found not only in the cultured cells, but in DN patients [16]. Therefore, Nrf2 may contribute to the improvement of inflammatory conditions such as DN.

With the onset of ROS production in diabetic kidneys, fibrosis is stimulated by increases in oxidative stress and inflammation. Protein kinase C (PKC) is associated with phosphorylation of serine/threonine residues in insulin receptors and is generated due to the synthesis of diacylglycerol (DAG) under the high intracellular concentration of glucose [17, 18]. In particular, PKC-*β*II, as one of the various isoforms of PKC, is well known to accelerate the pathogenesis of hyperglycemic kidney injuries, and it leads to insulin resistance as well as to dysfunction of various cells through the reduction of insulin receptor substrate- (IRS-) 2 tyrosine phosphorylation, resulting in defected insulin stimulation and intracellular accumulation of diacylglycerol in various organs [19, 20]. Thus, excessive production of PKC-*β*II in diabetic kidneys may induce formation of advanced glycation end products (AGE), as well as production of growth factors, such as transforming growth factor-*β* (TGF-*β*), connective tissue growth factor (CTGF), and vascular endothelial growth factor (VEGF) [21, 22].

Genistein, a class of phytoestrogens known as isoflavones, is mostly found in legumes. It has attracted attention because of its beneficial effects on prevention of metabolic disorders related to cardiovascular disease (CVD), obesity, cancer, and diabetes [23–26]. Thus, genistein has been extensively established as a multifunctional agent through enhancing the antioxidant defense system and anti-inflammation response. Recently, a study focused on the protective role of genistein on renal malfunction in rats fed a fructose rich diet, through the modulation of insulin resistance-induced pathological pathways [27]. Furthermore, Yuan et al. have noted that high doses of genistein (≥5 *μ*mol·L^−1^) protected renal mesangial cells against a hyperglycemic condition, which increased fibrosis through induction of fibrosis related genes, such as extracellular matrix (ECM) and TGF-*β* [28]. Another study has shown that genistein injections (10 mg/kg via i.p.) reduced urinary TBARs excretion and renal gp91phox expression, as well as decreased production of inflammatory markers, including p-ERK, ICAM-1, and MCP-1, in DN mice [29]. However, the efficacy of genistein on the connection of complex responses associated with oxidative stress and inflammation in DN is very uncertain. Moreover, little research has focused on the role of genistein in the development of DN in accordance with the degree of fasting blood glucose levels. In this study, we hypothesized that short-term genistein supplementation protects against diabetic kidney damage, depending on fasting blood glucose levels, through enhancement of hyperglycemia-induced oxidative stress, inflammation, and fibrosis in DN.

## 2. Materials and Methods

### 2.1. Animals

5.5-week-old female ICR mice were obtained from Daehan Biolink Co., LTD (Eumseong, Choungcheongbuk-do, Republic of Korea). Mice were individually housed in cages and acclimated for a week in animal facility conditions (22 ± 1°C and 50 ± 1% humidity with a 12 h in the light/dark). Diabetes was induced with a single intraperitoneal (i.p.) injection of 150 mg/kg alloxan monohydrate (Sigma-Aldrich Co., St Louis, MO, USA) in saline. On the other hand, nondiabetic control mice were injected with only saline in the same manner as the diabetic mice were treated. After a 1-week treatment, the induction of diabetes was confirmed by measuring fasting blood glucose levels. Fasting blood glucose levels from the mouse tail vein were measured by using a one-touch blood glucose meter (LifeScan Inc., Milpitas, USA). Fasting blood glucose levels ≥250 mg/dL were considered as diabetes. All mice care and experiments were approved by the Animal Care Institutional Committee of Kyung Hee University, Seoul, Republic of Korea.

### 2.2. Experimental Design

Diabetic mice were subdivided into two groups in accordance with fasting blood glucose (FBG) levels: medium high FBG (DMMH; 250 mg/dL ≤ FBG levels ≤ 450 mg/dL) and high FBG (DMH; 450 mg/dL ≤ FBG levels ≤ 600 mg/dL). Mice were treated with different diets and divided into the following groups (*n* = 9-10 per group): non-diabetic mice (CON) and diabetic-control mice (DMC; DMMH-C, DMH-C) mice were fed an AIN-93G diet without genistein supplementation (0%). DM-0.025% (0.025% genistein; DMMH-0.025%, DMH-0.025%) mice were fed 0.025% genistein (LC Laboratories, Woburn, MA) supplementation. DM-0.1% (0.1% genistein; DMMH-0.1%, DMH-0.1%) mice were fed 0.1% genistein supplementation. More details are shown in Table 1. At the end of the treatment (2 weeks), body weight, food consumption, and fasting blood glucose levels were measured once a week. Mice were fasted 8 h and anesthetized with isoflurane. Blood samples were collected by cardiac puncture, and then they were centrifuged at 3000 rpm for 10 min at 4°C. The kidneys were washed in saline and frozen immediately in liquid nitrogen. All samples were stored at −80°C until subsequent analysis.

### 2.3. Measurement of Serum Biochemical Analysis (Lipid Profile)

Blood samples were collected in heparin pretreated-tubes and centrifuged at 3000 rpm for 15 min to obtain plasma. The concentrations of total cholesterol (TC), triglyceride (TG), and high-density lipoprotein (HDL) cholesterol in plasma were assayed using the enzymatic method. Briefly, 20 *μ*L of plasma was mixed with an enzymatic kit (Bio-Clinical System, Gyeonggi-do, Republic of Korea) and incubated at 37°C water bath for 10 min. Concentrations were determined at 505 nm, 550 nm, and 500 nm, respectively.

The atherogenic index (AI) of plasma was calculated by the following ratio: (TC/HDL-C)/HDL-C.

### 2.4. Renal Function Monitoring

#### 2.4.1. Blood Urea Nitrogen (BUN) Measurement

Kidney function was measured by BUN. Specimens were examined by a commercially available kit (Asan pharmaceutical, Seoul, Republic of Korea) and incubated in a 37°C water bath for 5 min. Then, concentrations were determined at 580 nm using an ELISA reader (BIO-TEK instruments, Winooski, VT, USA).

#### 2.4.2. Plasma Creatinine

Plasma creatinine levels were examined by a creatinine assay kit (Bio-Clinical System, Gyeonggi-do, Republic of Korea) according to the manufacturer's protocol. Briefly, a mixture of plasma and picric acid were centrifuged at 3000 rpm for 10 min. Supernatant was reacted by an NaOH reagent at room temperature for 20 min and determined at 515 nm using an ELISA reader.

### 2.5. Malondialdehyde (MDA) Measurement in Kidneys

Malondialdehyde (MDA) measurement was usually used for estimation of lipid peroxidation levels [30]. Briefly, kidney homogenates were prepared in 0.15 M KCl buffer. A total of 200 *μ*L of homogenated kidney tissues were mixed with 200 *μ*L of 8.1% SDS and incubated at room temperature for 10 min. A total of 3 mL of 20% acetic acid-0.8% thiobarbituric acid (TBA) mixture (1 : 1, v/v) and 600 *μ*L of distilled water were added to make a total volume of 4 mL. The mixture was heated for 1 h in a 95°C water bath. After cooling in ice water, 1 mL distilled water and a 5 mL mixture of n-butanol and pyridine (15 : 1, v/v) were added to each tube. After centrifuging at 4000 rpm for 10 min, the upper layer was measured at 532 nm using an ELISA reader. Concentrations were determined using a 1,1,3,3-tetramethoxypropane (TMP, sigma-Aldrich, St. Louis, MO., USA) as a standard.

### 2.6. Preparation of Western Blot

For extraction of whole protein, 0.1 g of kidney tissues was homogenated at 4°C in lysis buffer (containing 20 mM Tris-HCl, 150 mM NaCl_,_ pH7.5, 1% NP40, 0.5% Na-deoxycholate stock, 1 mM EDTA, 0.1% SDS) with a protease inhibitor (Sigma Aldrich) and centrifuged at 14,000 rpm for 30 min. The resulting supernatants were frozen at 80°C until western blot analysis. Nuclear extracts were prepared from 0.25 g of kidney tissue and homogenated in 5 mL of buffer A (0.6% NP40, 150 mM NaCl, 10 mM HEPES (pH7.9), 1 mM EDTA, 0.5 mM PMSF, Leupeptin, Pepstatin, and Aprotinin). After centrifugation (2,000 rpm, 4°C, 30 sec), the supernatants incubated on ice for 5 min, centrifuged at 5,000 rpm for 5 min, and discarded the supernatant. 200 *μ*L of buffer B (25% Glycerol, 20 mM HEPES (pH7.9), 420 mM NaCl, 1.2 mM MgCl_2_, 0.2 mM EDTA, 0.5 mM dithiothreitol (DTT), 0.5 mM PMSF, Benzamidine, Leupeptin, Pepstatin, and Aprotinine) was added to the resulting pellet and shacked on ice for 20 min. The resulting suspensions were frozen at 80°C until western blot analysis. Protein concentration was measured using the NanoPhotometer (Implen, Germany). For gel electrophoresis, an equal amount of cytosolic and nuclear protein extracts (50 *μ*g and 25 *μ*g of total protein) was loaded in each lane. Proteins were separated by 10% SDS-PAGE and then transferred to the PVDF membrane (Millipore, Marlborogh, MA, USA). The membrane was blocked with 5% nonfat milk or 3% BSA in PBS containing Tween 20 (PBST) and probed overnight at refrigeration temperature with primary antibodies against Nrf2 (dilution 1 : 1000; Abcam), HO-1 (dilution 1 : 1000; Stressgen), GPx (dilution 1 : 16000; Abcam), CuZnSOD (dilution 1 : 1000; Santa Cruz Biotechnology), MnSOD (dilution 1 : 1000; Stressgen), p65 (dilution 1 : 200; Santa Cruz Biotechnology), pI*κ*B*α* (dilution 1 : 200; Santa Cruz Biotechnology), TNF-*α* (dilution 1 : 200; Santa Cruz Biotechnology), CRP (dilution 1 : 200; Abcam), MCP-1(dilution 1 : 1000; Cell Signaling), COX-2 (dilution 1 : 200; Stressgen), PKC (dilution 1 : 200; Santa Cruz Biotechnology), PKC-*β*II (dilution 1 : 200; Santa Cruz Biotechnology), TGF-*β*1 (dilution 1 : 200; Santa Cruz Biotechnology), and *β*-actin (dilution 1 : 1000; Santa Cruz Biotechnology). The membrane was washed with PBST and incubated with an HRP-conjugated secondary antibody (Santa Cruz Biotechnology, CA, USA) for 1 h. The target proteins were detected and visualized by enhanced chemiluminescence western blotting agents (Elpis Biotech, Republic of Korea) on an Image Analyzer (G box, Syngene, UK). The quantitation of each protein expression compared to the *β*-actin protein expression level was performed.

### 2.7. Statistical Analysis

All data are presented as mean ± SD. Sample normality was tested for primary outcomes (body weight, food intake, and fating blood glucose level). Statistical differences of variables (body weight, food intake, and fasting blood glucose level) between CON and DMH-C were analyzed by unpaired *t*-test. The effects of DM severity (normal control, DMMH, and DMH) and/or genistein supplemented diet (0, 0.025, and 0.1%) were analyzed by one-way analysis of variance (ANOVA). Two-way ANOVA was used to analyze the effects of the genistein supplemented diet and DM severity and their interaction on outcomes followed by post hoc test (Tukey HSD) using SPSS (20.0 K for Windows) statistical analysis program. For all outcomes, a value of *P* < 0.05 was considered significant.

## 3. Results

### 3.1. Effect of Genistein Supplementation on Body Weight and Food Intake

**Figure 1:**
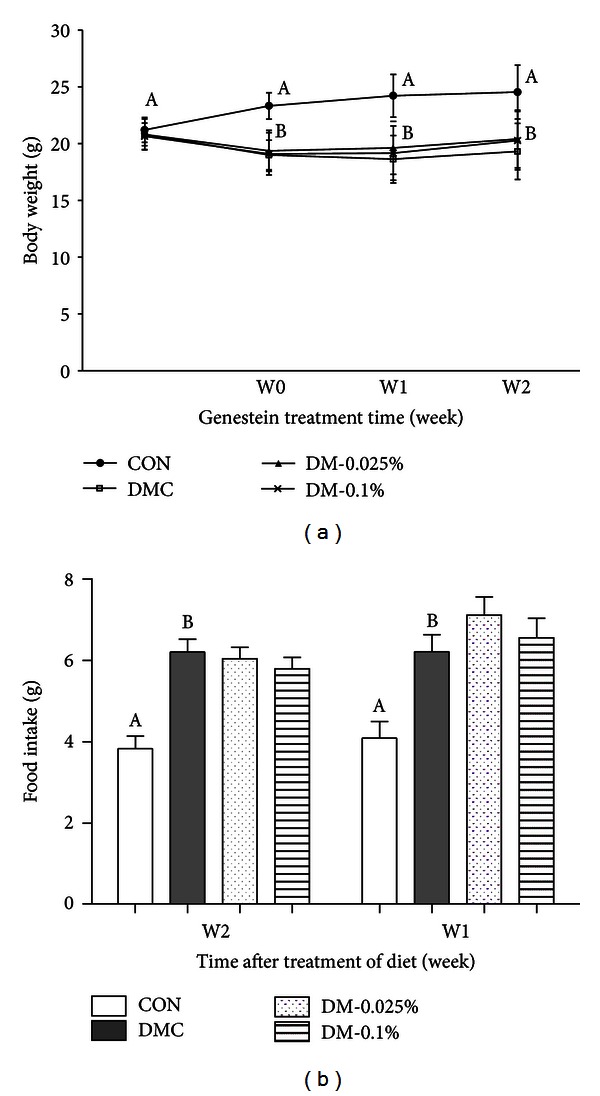
Effect of genistein supplementation on body weight (a) and food intake (b) in experimental mice. Data are presented as means ± SD (*n* = 9-10/group). Mean values with different letters were significantly different, *P* < 0.05. Statistical differences of variables between CON and DMH-C analyzed by unpaired *t*-test were shown in capital letters. CON, control mice; DMC, diabetic control mice; DM-0.025%, diabetic mice supplemented with 0.025% genistein; DM-0.1%, diabetic mice supplemented with 0.1% genistein.

Changes in body weight and food intake during the experimental period are shown in Figure 1. After 1 week of alloxan injection to induce diabetes, body weight in diabetic mice was significantly lower than that of CON (Figure 1(a)). However, genistein supplementation, regardless of dose, did not prevent the decrease in body weight. Food intake was significantly increased in diabetic mice, regardless of genistein supplementation compared with the CON group (Figure 1(b)).

### 3.2. Effect of Genistein Supplementation on Changes in Fasting Glucose Level

**Figure 2:**
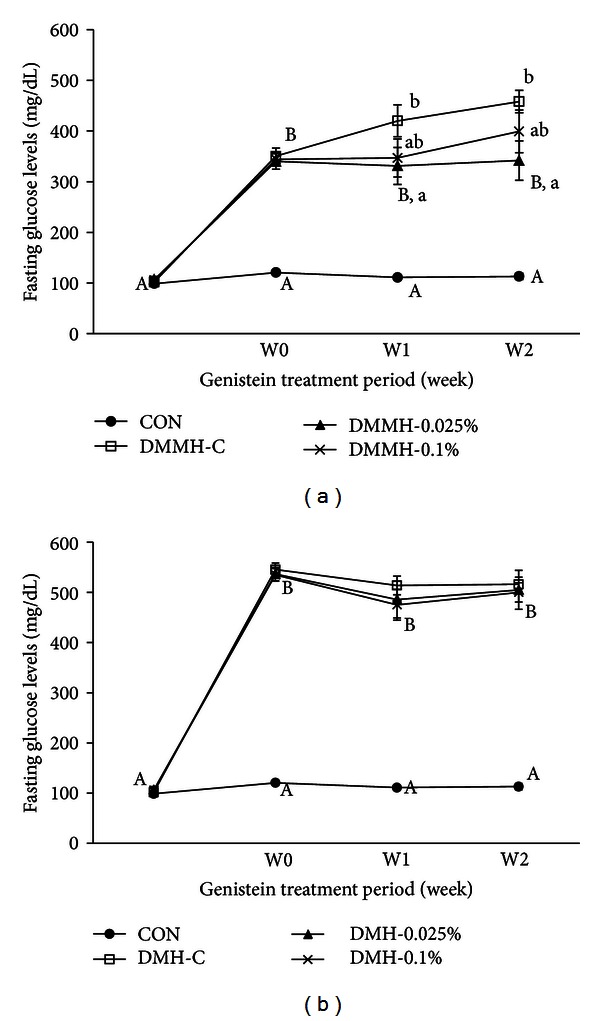
Effect of genistein supplementation on fasting blood glucose levels in experimental mice. Fasting blood glucose levels in DMMH group (a) and fasting blood glucose levels in DMH group (b). Data are presented as means ± SD (*n* = 9-10/group). Mean values with different letters were significantly different, *P* < 0.05. Statistical differences of variables between CON and DMH-C analyzed by unpaired *t*-test were shown in capital letters and effects of the genistein supplemented diets on body weight and food intake in diabetic mice using one-way ANOVA was represented by small letters.

Levels of fasting blood glucose were significantly higher in all the diabetic groups compared to the CON group. The 0.025% genistein supplementation in DMMH significantly decreased FBG levels, but 0.1% genistein in DMMH did not significantly reduce FBG levels (Figure 2(a)). In Figure 2(b), genistein supplementation in DMH did not show a difference in FBG levels.

### 3.3. Effect of Genistein Supplementation on Biochemical Markers

#### 3.3.1. Lipid Profiles

To examine the effect of genistein supplementation on lipid profiles, we measured plasma lipid profiles. As shown in Table 2(a), plasma levels of total cholesterol (TC) and triglycerides (TG) were elevated in the DMC more than in the CON, but there were no significant differences between the DMC groups and genistein supplementation groups. Moreover, the plasma level of high density lipoprotein cholesterol (HDL-C) did not differ among the groups. Thus, the DMC groups were characterized by a markedly elevated atherogenic index (AI) as compared to the CON group, but genistein supplementation did not effectively decrease AI.

#### 3.3.2. Blood Urea Nitrogen (BUN)

As shown in Table 2(b), the concentration of BUN was significantly increased in the DMMH-C and DMH-C groups compared to that of the CON group (*P* < 0.05). BUN concentrations of 0.025% DMMH and 0.1% DMMH were decreased by 47% and 43%, respectively, as compared to the DMMH-C group, BUN concentrations of 0.025% DMH and 0.1% DMH groups were significantly decreased by 52% and 51%, respectively, as compared to the DMH-C group.

#### 3.3.3. Plasma Creatinine

As shown in Table 2(c), plasma creatinine levels were significantly elevated in the DMMH-C and the DMH-C groups compared with the CON group (*P* < 0.05). The concentration of plasma creatinine was much higher in the DMH-C group than in the DMMH-C group. Genistein supplementation, regardless of dose, in the DMMH group ameliorated plasma creatinine levels. However, although the plasma creatinine level of the DMH group reached more than 1.5-fold compared to the CON group, only the 0.025% genistein supplementation significantly decreased plasma creatinine levels in DMH.

#### 3.3.4. MDA

To examine the effect of genistein supplementation on oxidative stress in kidneys, kidney MDA levels were measured. MDA levels were significantly elevated in both the DMMH-C and DMH-C groups (1.5-fold above CON, *P* < 0.05). On the other hand, genistein supplementation in the DMMH-C groups reduced the level of MDA concentration to the normal level. The supplementation of 0.025% genistein significantly decreased the kidney MDA levels (DMMH-L; 33.26%, DMH-H; 29.22% compared to DMC), but the supplementation of 0.1% genistein did not significantly reduce it in the DMH mice (Table 2(d)).

### 3.4. Effect of Genistein on Protein Expression Levels of Oxidative Stress Markers in Diabetic Kidneys

**Figure 3:**
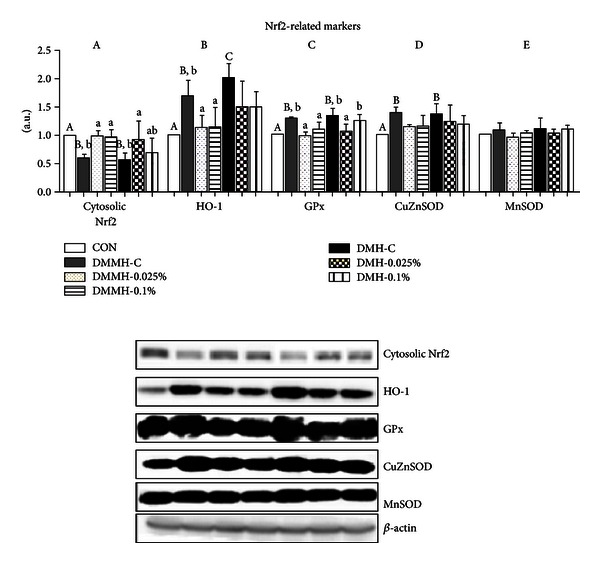
Effect of genistein supplementation on the kidney protein levels of cytosolic Nrf2 (a), HO-1 (b), GPx (c), CuZnSOD (d), and MnSOD (e) in experimental mice. All results were conducted at least three times. Data are presented as means ± SD (*n* = 9-10/group). Mean values with different letters were significantly different, *P* < 0.05. Statistical differences of variables among CON, DMMH-C, and DMH-C analyzed by one-way ANOVA were shown in capital letters and effects of the genistein supplemented diet and/or DM severity using two-way ANOVA were represented by small letters.

We performed western blot analysis to determine whether genistein supplementation declined the activation of Nrf2-linked oxidative stress proteins in DN. The levels of cytosolic Nrf2 protein expression decreased in the DMMH-C and DMH-C groups (*P* < 0.05, Figure 3(a)). We found that the reduction of cytosolic Nrf2 protein levels in DMMH was effectively restored by genistein supplementation regardless of dose. The 0.025% genistein supplementation in DMH significantly raised the cytosolic Nrf2 levels, more than the DMH-C (*P* < 0.05), but 0.1% genistein in DMH did not significantly affect cytosolic Nrf2 expression. The expression of HO-1 levels, a representative target gene in the Nrf2 pathway, was significantly increased in the DMC as compared with the CON (*P* < 0.05). In addition, the expression of HO-1 was much higher in the DMC-C group than in the DMMH-C group. Genistein supplementation, regardless of dose, completely reduced the expression of HO-1 levels in DMMH and DMH (Figure 3(b)). Furthermore, GPx levels were significantly increased in DMC mice, more so than in CON mice (Figure 3(c)). GPx expression in the DMMH group was normalized by genistein supplementation independently of the dose. In the DMH group, 0.025% genistein supplementation significantly reduced GPx expression, while 0.1% genistein supplementation was not changed. As shown in Figure 3(d), the expression of CuZnSOD levels was higher in the DMMH-C and the DMH-C than in the CON. Genistein supplementation relatively decreased the CuZnSOD levels, although the difference was not statistically significant. Unfortunately, the expression of MnSOD levels did not significantly differ among the groups (Figure 3(e)).

### 3.5. Effect of Genistein on Protein Expression Levels of Inflammation Markers in Diabetic Kidneys

**Figure 4:**
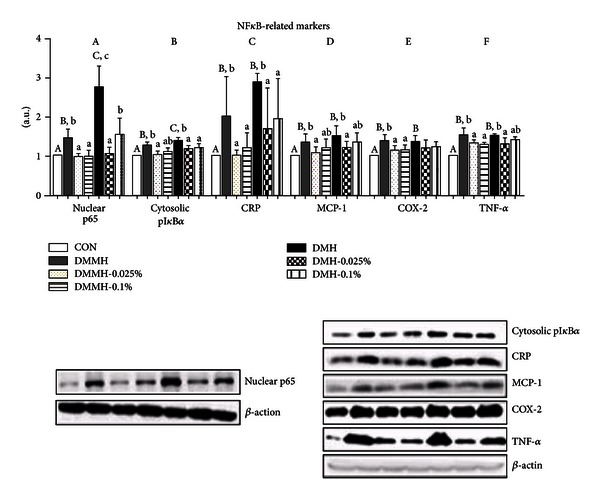
Effect of genistein supplementation on the kidney protein levels of p65 (NF*κ*B) (a), pI*κ*B*α* (b), CRP (c), MCP-1 (d), COX-2 (e), and TNF-*α* (f) in experimental mice. All results were conducted at least three times. Data are presented as means ± SD (*n* = 9-10/group). Mean values with different letters were significantly different, *P* < 0.05. Statistical differences of variables among CON, DMMH-C, and DMH-C analyzed by one-way ANOVA were shown in capital letters and effects of the genistein supplemented diet and/or DM severity using two-way ANOVA were represented by small letters.

**Figure 5:**
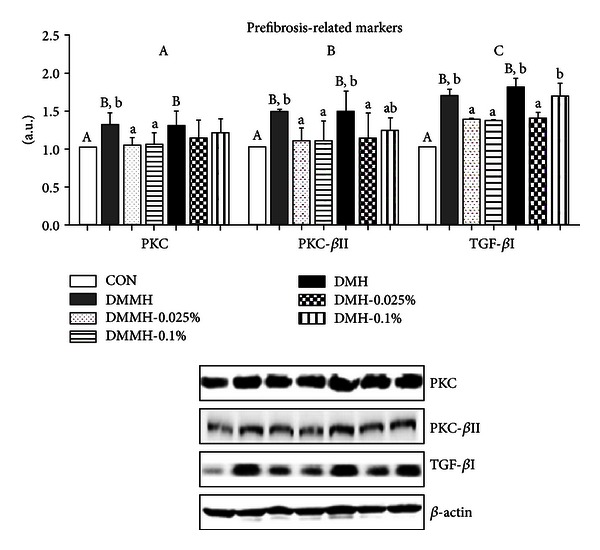
Effect of genistein supplementation on the kidney protein levels of PKC (a), PKC-*β*II (b), and TFG-*β*I (c) in experimental mice. All results were conducted at least three times. Data are presented as means ± SD (*n* = 9-10/group). Mean values with different letters were significantly different, *P* < 0.05. Statistical differences of variables among CON, DMMH-C and DMH-C analyzed by one-way ANOVA were shown in capital letters and effects of the genistein supplemented diet and/or DM severity using two-way ANOVA was represented by small letters.

We tested to elucidate whether the genistein supplementation reduced the expression of NF*κ*B-related inflammatory proteins in DN. The levels of NF*κ*B (p65) and pI*κ*B*α*, an indirect marker for measuring the activation of NF*κ*B, were significantly increased in the DMC as compared to the CON (Figures 4(a) and 4(b)). Genistein supplementation, regardless of dose, significantly decreased the levels of cytosolic pI*κ*B*α* and nuclear NF*κ*B in DMMH and DMH compared to DMMH-C and DMH-C (*P* < 0.05). Next, we measured the expression of CRP, which was increased by alloxan-induced diabetes (Figure 4(c)). Genistein supplementation, regardless of dose, significantly inhibited increased CRP levels in the DMMH and DMH groups (*P* < 0.05). As shown in Figure 4(d), MCP-1 levels were higher in DMMH-C and DMMH-C than in CON, and the levels in DMMH and DMH were markedly lower by 0.025% genistein. However, 0.1% genistein showed no significant inhibitory effects on the MCP-1 levels in DMMH and DMH. The protein expression of COX-2, as a representative marker of the NF*κ*B-pathway, was significantly elevated in DMC (*P* < 0.05). Genistein supplementation in DMMH suppressed the upregulation of COX-2 levels, while there was no difference in the DMH groups (Figure 4(e)). Additionally, TNF-*α* levels were significantly higher in all diabetic mice than in CON mice (Figure 5(f)). However, the genistein supplementation groups exhibited a remarkable reduction in the expression of TNF-*α* in comparison with the DMC groups, excluding DMH-0.1% (*P* < 0.05).

### 3.6. Effect of Genistein on Protein Expression Levels of Fibrosis-Mediated Markers in Diabetic Kidneys

We examined the question as to whether genistein supplementation contributed to enhancing an antidiabetic kidney fibrosis pathway in the experimental mice. Our data showed a significant increase in PKC and PKC-*β*II protein expression in the DMC groups, regardless of FBG levels (Figures 5(a) and 5(b)). The levels of PKC and PKC-*β*II protein expression in DMMH were effectively decreased by genistein supplementation regardless of dose (*P* < 0.05). The level of PKC expression in DMH was reduced by genistein supplementation, regardless of dose, but there was not a significant difference (Figure 5(a)). The 0.025% genistein supplementation in DMH was more effective at reducing the level of PKC-*β*II protein expression than the 0.1% genistein in DMH (Figure 5(b)). To further investigate the mechanism of an antifibrosis effect of genistein, we tested the expression of TFG-*β*I. TFG-*β*1, as one of the most potent fibrogenic response markers, was greater in DMC than those of CON. However, as contrasted with nontreated genistein supplementation, genistein supplementation groups experienced significant decreased expression of TFG-*β*I, except DMH-0.1% (*P* < 0.05).

## 4. Discussion

The present study provides some good evidence that genistein has an ability to protect kidneys from hyperglycemia-induced oxidative stress, inflammation, and fibrosis in alloxan-induced diabetic mice. Although genistein has beneficial influences with respect to both antioxidative stress and anti-inflammation [31], there is no clear underlying mechanism by which genistein can boost a protective role in DN progression in accordance with blood glucose levels. A severe loss of body weight (B.W.) and an increase of food intake generally occur in diabetic conditions [32–34]. We also examined the question as to whether genistein supplementation did not improve body weight loss and food intake as shown in the previous studies [35, 36].

An ultimate treatment goal of diabetes and its complications is the control of the FBG levels [37]. According to previous studies, a glucose level with 250–450 mg/dL was diagnosed as mild hyperglycemia [38], and a glucose level above 450 mg/dL was considered as severe hyperglycemia [39]. In this study, diabetes with different FBG levels, in a range from 250 mg/dL to 600 mg/dL (maximum read by commercial glucometer), and FBG levels of 450 mg/dL were used as the criteria of hyperglycemia classification. Our data found that genistein supplementation decreased the FBG level in DMMH mice, but it did not affect blood glucose levels in DMH mice. Previously, genistein has been shown to have an effect on the modulation of blood glucose levels *in vivo*, regardless of the manner of genistein administration and treatment period and dose, which have included short-term (for 16 days) i.p. injection of genistein (1 mg/kg B.W./day) in rats fed a fructose rich diet [27] and long-term (for 9 weeks) dietary supplementation of genistein (0.02% w/w) in nonobese diabetic (NOD) mice [35]. In other findings [40], it was discovered that not a low dose (under 15 mg/kg B.W.) but a high dose (15–30 mg/kg B.W.) of genistein supplementation markedly reduced blood glucose levels in alloxan-induced diabetes mice. However, researchers have not proven a potential benefit of genistein on diabetic animals with different levels of FBG. Collectively, the results suggested that short-term supplementation of genistein possesses the capacity to reduce hyperglycemia in the DMMH group without insulin treatment but not in DMH group.

Impairment of insulin secretion in diabetes increases the release of free fatty acids (FFA) into the liver, and it may cause an increase in triglyceride production [41]. It promotes diabetic dyslipidemia, which may worsen the interplay of inflammation and intrarenal fibrosis [42, 43]. A previous study reported that genistein supplementation (600 mg/kg diet) for 3 weeks improved plasma lipid profiles (TC, TG, and HDL) in diabetic mice [44], whereas another study confirmed that genistein supplementation (250 mg/kg diet) for 4 weeks did not improve the plasma lipid profiles in diabetic mice [45]. Our data showed that genistein supplementation, regardless of supplementation doses (0.025% in a 250 mg/kg diet or 0.1% in a 1000 mg/kg diet), did not show a lowering effect on dyslipidemia. These results suggest that diabetes-related dyslipidemia is controlled by a relatively high concentration of genistein supplementation for longer than 3 weeks of treatment.

BUN and plasma creatinine, as waste products of metabolism, preannounce damage in kidney function [46]. We observed that BUN and plasma creatinine levels were increased in DMC mice, especially in DMH-C. Sung et al. [47] reported that the genistein addition (10 mg/kg B.W.) for 3 days significantly reduced BUN and serum creatinine levels in cisplatin-induced acute renal injury. We also observed that genistein supplementation decreased BUN levels in DMMH group and DMH group. BUN is usually done together with a plasma creatinine, which is a more sensitive marker of kidney damage. Genistein supplementation in DMMH alleviated plasma creatinine levels to normal levels and significantly reduced the levels in DMH-0.025%, but not in DMH-0.1%. Thus, our data demonstrated that genistein, regardless of supplemented dose, could prevent an impairment of kidney function in DMMH, and only the 0.025% genistein supplementation may have beneficial effects on kidney damage when the FBG level is very high.

In diabetic conditions, a continuous overproduction of ROS and an antioxidant defense system may cause mitochondrial impairment [48]. Thus, oxidative stress is considered as a mediator in tissue injury, including liver, brain, and kidney. The kidney is known as a highly sensitive organ in oxidative stress conditions because lipid composition in kidneys comprises long-chain polyunsaturated fatty acids [49]. In our experiments, the MDA accumulation was increased by consequences of oxidative stress, such as diabetes [50], but genistein (6 mg/kg/B.W.) decreased the MDA levels in the brain and liver of STZ-induced diabetic mice [51]. Our study also observed that genistein supplementation significantly lowered kidney MDA levels in diabetic mice, except in DMH-0.1%. A previous study demonstrated that a high dose of genistein can have adverse actions as a prooxidant, depending on the status of oxidative stress [52]. Therefore, the results suggest that 0.1% genistein supplementation may act as a prooxidant in the DMH group, which is considered as possessing higher oxidative stress status compared to the DMMH group.

Nrf2 is normally combined with its repressive protein Keap1 (Kelch-like ECH-associated protein-1) in cytoplasm [53]. In an oxidative stress state, Nrf2 is separated from Keap1 and translocated to the nucleus. It activates antioxidant enzymes such as HO-1, GST, NADH(H) quinoline oxidoreductase-1 (NQO1), and glutathione peroxidase (GSH-Px) [54, 55]. Therefore, Nrf2 and its downstream genes play a crucial role in defense of cellular damage against oxidative stress, but its overproduction may lead to paradoxical effects in connection with a disturbance in the protection of cells from oxidative damage [56]. Previous studies reported that expression of Nrf2 and antioxidant genes, such as HO-1, SOD, catalase (CAT), and GPx, was increased in diabetes [57–59]. The results suggest that excessive production of oxidative stress seems to stimulate increases in antioxidant enzyme production in order to eliminate oxidative stress agents in DM. It is known that genistein has cytoprotective effects on Nrf2 activation and its downstream antioxidant enzymes, including HO-1, SOD, CAT, and GSH [60]. Our data showed that cytosolic Nrf2 was decreased in diabetes, which may lead to an increase in nuclear Nrf2 activation as a consequence of the activation of a cellular antioxidant defense with increased transcription of antioxidant genes. The results were reversed by the genistein supplementation, and this outcome supports the hypothesis that genistein supplementation was able to reestablish the cell homeostasis. However, 0.1% genistein in DMH did not markedly change Nrf2 levels. These findings indicate that 0.1% genistein may be not enough to provide beneficial effects on the Nrf2-mediated oxidative stress pathway in diabetic mice with high FBG levels.

HO-1, a representative marker of an Nrf2-related stress response, has been found to increase in pathological conditions such as diabetes [61–63]. The present study demonstrated that genistein supplementation, regardless of dose, tends to reduce the expression of HO-1 levels in DMMH and DMH. Moreover, protein levels of GPx and SOD isoforms are associated with oxidative damage and mitochondrial dysfunction through hydrogen peroxide (H_2_O_2_) production by the glucose oxidase system [64–66]. A previous study [67] demonstrated that GPx activity was increased in diabetic mice organs including the liver, pancreas, and kidney. Our findings proved that genistein supplementation reduced GPx levels, except the DMH-0.1% group. However, genistein did not significantly reduce CuZnSOD levels and did not change MnSOD levels among the groups. These results suggest that genistein supplementation selectively alleviated oxidative stress through the regulation of Nrf2 levels and its consequent events. Moreover, the DMH group with a high dose supplementation of genistein may have more oxidative stress.

Nrf2-mediated interplay has two sides of action as either a regulator of antioxidant response or a reactive promoter of oxidative stress in abnormal conditions [68]. On the basis of our results, we proposed that an overproduction of reactive oxygen species (ROS) in diabetes can trigger activation of nucleus Nrf2 and transcription of its downstream target enzymes. On the other hand, oxidative stress leads to activation of the inflammatory-mediated transcription factor, NF*κ*B. Thus, we identified the fact that genistein supplementation attenuated the hyperglycemia-induced inflammatory responses through the regulation of the NF*κ*B pathway. Many studies have reported experimental evidence showing that NF*κ*B was activated in diabetic kidneys [69, 70], and genistein supplementation (1 mg/kg/B.W.) attenuates NF*κ*B (P65) activation in kidneys of rats fed a fructose rich diet [71]. We have investigated the pI*κ*B*α* level in cytosol and NF*κ*B (p65) level in nucleus to identify NF*κ*B activation. pI*κ*B*α* level is a representative of NF*κ*B activation in cytosol because pI*κ*B*α* after phosphorylation of I*κ*B*α* is subsequently ubiquitinated and degraded via the proteasome pathway [72]. NF*κ*B, p65 and p50 heterodimer, separated from I*κ*B*α* is translocated into the nucleus and activates the expression of inflammatory genes. Our data showed that the protein levels of pI*κ*B*α* in cytosol and NF*κ*B in nucleus as increased in DMC and lowered in genistein supplementation. These results imply that genistein supplementation blocked NF*κ*B activation by reduction of pI*κ*B*α*.

Activation of the NF*κ*B signaling pathway is known to enhance inflammatory cytokines (IL-1*β*, TNF-*α*) and activate fibrosis markers (AGE, RAGE) in diabetic mice [73]. Among them, TNF-*α* (tumor necrosis factor-*α*) is the main proinflammatory cytokine, which acts toward the progression of diabetic kidney disease through recruitment of macrophages and neutrophils into the kidney [74]. An increased renal TNF-*α* level is correlated with indicators of renal failure in DM animals [75] and patients [76]. The present study confirmed that TNF-*α* levels in genistein supplementation groups were even lower than those in DMC groups. However, DMH with 0.1% genistein did not show significant differences, which means that 0.025% genistein is more effective than 0.1% genistein for DN with high FBG levels. CRP is generally increased in inflammatory conditions, such as those found in DN patients and animals [77, 78]. Dietary isoflavone, including genistein, has a capacity to decrease the concentration of CRP in human plasma [79, 80]. Similarly, the expression of CRP levels in DN mice was significantly reduced in all diabetic mice supplemented with genistein, more so than those of DMC. In addition, expression of MCP-1 and COX-2 is relevant to NF*κ*B-mediated modulation of an inflammatory cascade, which contributes to endothelial dysfunction [81–83]. Genistein (10 mg/kg via i.p., three times a week) as a tyrosine kinase inhibitor has been shown to reduce significantly the excretion of urinary MCP-1 in STZ-induced diabetic mice [29]. Our results showed that the production of MCP-1 significantly decreased in the 0.025% genistein supplementation groups, whereas the 0.1% genistein supplementation groups did not reduce MCP-1 production. Thus, the results suggest that a relatively low dose of genistein may reduce MCP-1 protein via inhibition of NF*κ*B activation. Moreover, genistein, as an inhibition agent of cell proliferation, inhibited COX-2 protein in cancer cells [84], a result that improved the balance of angiogenesis and apoptosis. In our findings, overproduction of COX-2 in DMMH was attenuated by genistein supplementation at both 0.025% and 0.1% levels, but not in DMH. In other words, 0.025% genistein supplementation in diabetes with medium high FBG may control vascular homeostasis through suppression of NF*κ*B-mediated inflammation.

Moreover, the findings indicate that Nrf2 activation and its downstream signalling pathway interact with the activation of NF*κ*B-mediated inflammatory responses in diabetes, and genistein supplementation might reduce activation of antioxidant defence systems and inflammatory responses by regulation of Nrf2 and NF*κ*B interactions.

Nrf2 and NF*κ*B interactions may play a serious role in fibrosis in diabetic kidneys, which corresponds with increased PKC-mediated pathways in hyperglycemic conditions. This conclusion has been supported by several *in vitro* experiments [85], which demonstrated that genistein (40 *μ*M) blocked PKC activation in VEGF-stimulated endothelial cells [86]. However, there is no research focusing on the effect of genistein on PKC inhibition in diabetic animals. The PKC-*β* isoform is mainly responsible for hyperglycemia-induced fibrosis in DN [87]. Several reports have provided evidence that genistein attenuated the levels of PKC isoenzymes, such as PKC-*β*I, in rat ventricular monocytes [88], as well as levels of PKC-*β*II in rats fed a fructose rich diet, an experiment that constitutes a hypertension mouse model [89]. Our data showed the different effects of genistein on prefibrosis-related markers, both PKC and PKC-*β*II, in DMH depending on their treatment dose. The 0.1% genistein supplementation in the DMH group did not significantly reduce the levels of both PKC and PKC-*β*II. This result suggests that 0.1% genistein supplementation may not have beneficial effects on fibrosis in diabetes with high FBG. Continuous exposure of ROS in hyperglycemia may also lead to changes in cell membrane structure. The transforming growth factor *β*I (TFG-*β*I), a family of fibrogenic cytokine, has been generally known to induce deposition of matrix components, such as ECM, as well as synthesis of glomerulosclerosis in DN rats [90]. A hyperglycemic condition induces an increase in TGF-*β*I levels, and it stimulates fibrosis of numerous organs, such as the kidney [91]. Thus, inhibition of TGF- *β*I is a key player in protection of diabetic kidneys. Genistein has been proven effective in the prevention of hyperglycemia-induced fibrosis by inhibiting the expression of TGF- *β*I [28] and TGF- *β*II [92]. In particular, the data showed that genistein was able to inhibit TGF-*β*I production, not at a low concentration (≤5 *μ*mol·L^−1^), but at a high concentration (≥5 *μ*mol·L^−1^) [28], and to reduce TGF-*β*II production at the high concentration level (5 *μ*g/mL) [92]. However, our results demonstrated that 0.1% genistein supplementation did not protect against the fibrosis process, represented by TGF-*β*II, from a severe hyperglycemic condition in DMH. The results might be associated with the prooxidant effect of genistein at high doses on severe hyperglycemia as a promotor of prefibrosis in DN.

Taken together, our data evidenced that genistein supplementation inhibited hyperglycemia-induced fibrosis pathways as well as the activation of the transcription factors, Nrf2 and NF*κ*B. Moreover, we found that genistein supplementation has selective effects on diabetic kidney damage in accordance with FBG levels. In previous studies, genistein has been shown to have adverse effects in pathogenetic conditions, which may act as prooxidants and accelerate the progression of disease [93]. However, this study has several limitations. Only the short-term effects of dietary genistein supplementation have been investigated with respect to diabetes induced kidney damage. Long-term supplementation protocols may be helpful to verify the role of genistein in the DMH group (>450 mg/dL) because the DMH group may need a longer time to control inflammation, oxidative stress, and fibrosis processes. Moreover, short-term supplementation at different doses did not change plasma lipid profiles. This result might be associated with supplemented doses and periods, as well as FBG levels. In addition to treatment regimens, histological analysis of kidneys may be more helpful to investigate fibrosis process in this study.

In conclusion, understanding the molecular mechanisms that regulate oxidative stress, inflammation and fibrosis is critical not only in diabetic kidney damage, but also in other diabetic complications. Hence, the results of this study may provide critical insight into future nutritional intervention strategies, with or without insulin treatment, designed to prevent diabetic complications according to FBG levels.

## Figures and Tables

**Table 1:** Classification of experimental groups.

Group	Treatment
CON	Nondiabetic mice were fed AIN-93G diet without genistein supplementation
DMMH-C	Diabetic-control mice with the level of medium-high FBG between 250 and 450 were fed AIN-93G diet without genistein supplementation
DMMH-0.025%	Diabetic mice with medium high FBG levels between 250 and 450 were fed 0.025% genistein supplementation
DMMH-0.1%	Diabetic mice with medium high FBG levels between 250 and 450 were fed 0.1% genistein supplementation
DMH-C	Diabetic-control mice with the level of high FBG between 450 and 600 did not receive genistein supplementation
DMH-0.025%	Diabetic mice with high FBG levels between 450 and 600 were fed 0.025% genistein supplementation
DMH-0.1%	Diabetic mice with high FBG levels between 450 and 600 were fed 0.1% genistein supplementation

**Table 2:** Effect of genistein supplementation on biochemical markers.

Group	Lipid profiles				Kidney function		Oxidative stress
	TC (mg/dL)	TG (mg/dL)	HDL-C (mg/dL)	AI	BUN (mg/dL)	Creatinine (mg/dL)	MDA (nM)
CON	121.72 ± 12.83^A^	87.03 ± 19.35^A^	77.74 ± 13.45	0.57 ± 0.23^A^	17.76 ± 3.04^A^	0.57 ± 0.08^A^	16.87 ± 4.28^A^
DMMH-C	187.98 ± 21.15^B^	119.89 ± 26.92^AB^	70.24 ± 11.61	0.97 ± 0.2^B^	46.99 ± 21.49^BCb^	0.81 ± 0.13^Bb^	25.82 ± 6.23^Bb^
DMMH-0.025%	171.65 ± 21.47	96.19 ± 22.39	75.93 ± 5.62	0.80 ± 0.14	24.93 ± 10.99^a^	0.62 ± 0.10^a^	17.23 ± 3.01^a^
DMMH-0.1%	178.56 ± 39.68	109.65 ± 14.96	73.81 ± 5.36	0.88 ± 0.24	26.78 ± 5.56^a^	0.64 ± 0.09^a^	17.65 ± 2.97^a^
DMH-C	206.64 ± 43.56^B^	132.21 ± 20.88^B^	64.05 ± 8.78	0.98 ± 0.22^B^	67.35 ± 58.51^Cb^	0.94 ± 0.25^Cc^	25.90 ± 3.57^Bb^
DMH-0.025%	172.29 ± 45.89	107.71 ± 29.20	73.83 ± 15.64	0.93 ± 0.23	32.30 ± 10.68^a^	0.72 ± 0.11^ab^	18.33 ± 5.26^a^
DMH-0.1%	173.32 ± 46.33	121.43 ± 35.15	71.10 ± 29.38	0.94 ± 0.41	32.78 ± 7.95^a^	0.79 ± 0.21^bc^	20.04 ± 4.94^ab^

Abbreviations: TC: total cholesterol, TG: triglyceride, HDL: high density lipoprotein cholesterol, AI: atherogenic index, BUN: blood urea nitrogen, and MDA: malondialdehyde.

Mean values with different letters were significantly different (*P* < 0.05). Statistical differences of variables among CON, DMMH-C, and DMH-C analyzed by one-way ANOVA were shown in capital letters and effects of the genistein supplemented diet and/or DM severity using two-way ANOVA were represented by small letters.

## Abbreviations

DN: Diabetic nephropathy
FBG: Fasting blood glucose
ROS: Reactive oxygen species
BUN: Blood urea nitrogen
NF*κ*B: Nuclear factor kappa B
Nrf2: Nuclear related factor E2
HO-1: Heme oxygenase-1
CRP: C-reactive protein
MCP-1: Monocyte chemotactic protein-1
COX-2: Cyclooxygenase-2
GPx: Glutathione peroxidase
MDA: Malondialdehyde
CuZnSOD: Copper zinc superoxide dismutase
MnSOD: Manganese superoxide dismutase
pI*κ*B*α*: Phosphorylated inhibitory kappa B alpha
TNF-*α*: Tumor necrosis factor-alpha
PKC: Protein kinase C
PKC-*β*II: Protein kinase C-beta II
TGF-*β*I: Transforming growth factor-beta I.
